# Effect of Morbidities, Depression, Anxiety, and Stress on Oral Health-Related Quality of Life among Migrant Elderly Following Children in Weifang, China

**DOI:** 10.3390/ijerph19084677

**Published:** 2022-04-13

**Authors:** Hexian Li, Fanlei Kong

**Affiliations:** 1 Centre for Health Management and Policy Research, School of Public Health, Cheeloo College of Medicine, Shandong University, Jinan 250012, China; 202036426@mail.sdu.edu.cn; 2 NHC Key Lab of Health Economics and Policy Research, Shandong University, Jinan 250012, China

**Keywords:** distress, depression, anxiety, oral health-related quality of life, migrant elderly following children

## Abstract

This study explored the relationship between depression, anxiety, stress, morbidity, and oral health-related quality of life (OHRQoL) in the migrant elderly following children (MEFC) in Weifang, China. A total of 613 MEFC were selected using multistage cluster random sampling. The GOHAI scale was used to evaluate oral health-related quality of life. The DASS-21 scale was used to assess levels of depression, anxiety, and stress. Univariate analysis and binary logistic regression were used to analyze the correlation between these indicators and oral health-related quality of life, of which 43.9% were classified as having poor oral health. Logistic regression analysis showed that the MEFC who were of older age (OR = 0.965, *p* = 0.039), with hypertension (OR = 0.567, *p* = 0.004), with gastroenteropathy (OR = 0.263, *p* = 0.007), had received an outpatient service in the past year (OR = 0.669, *p* = 0.048), were depressed (OR = 0.338, *p* = 0.012), and anxious (OR = 0.414, *p* = 0.026) were less likely to report good oral health status. On the other hand, the MEFC with a high school education or above (OR = 1.872, *p* = 0.020) were more likely to report good oral health than those with primary school education and below. In conclusion, with regard to depression, anxiety, and stress: the results indicated that the fewer morbidities, the lower the level of depression and anxiety and the better the OHRQoL of MEFC. Targeted measures for government, communities, and family members were given to improve the OHRQoL of MEFC.

## 1. Introduction

With China’s aging population and accelerating urbanization, a large number of young people are leaving their hometowns and choosing to settle elsewhere. Subsequently, the parents follow their children to live in other areas, resulting in a growing number of migrant elderly following children (MEFC) [1]. The China Mobile Population Development Report (2018) shows that the number of elderly mobile population in China continues to increase. By 2015, the elderly mobile population in China reached 13.04 million, accounting for 5.3% of the national mobile population [2]. In this study, MEFC refers to those who leave the familiar rural or urban environment and follow their children to live in big cities to take care of their family members or provide for the aged [3].

The number of older migrant adults continues to increase, and therefore promoting their health and quality of life is an important component of public health [4]. However, basic public health services are underutilized by older migrants as compared with the general population [5]. Since the Hukou System is one of China’s major administrative systems, it is a major barrier to socioeconomic mobility and can exacerbate health inequalities [6]. Older immigrants are often not entitled to the same social welfare and health services as local residents; therefore, physical and mental health problems are more likely to appear in them [7,8,9]. Oral health is a critical element of an individual’s overall health and closely related to maintaining and improving the quality of life of older adults [10]. The World Health Organization has shown that the negative impact of poor oral conditions on the quality of life of older adults is a major public health concern [11]. However, oral health has been neglected for a long time in China’s overall health agenda, and oral diseases are of increasing concern [12]. Therefore, the oral health of MEFC should be considered seriously.

Oral health-related quality of life (OHRQoL) is “a multidimensional construct that reflects (among other things) people’s comfort when eating, sleeping, and engaging in social interaction; their self-esteem; and their satisfaction with respect to their oral health” [13]. Older adults have more oral health problems, owing to their health burden and increased tooth retention. Poor oral health-related quality of life can damage a person’s mental state [14], social relationships [15], and physical health [16,17]. Research has found that immigration status and lower adaptive ability were associated with poorer self-assessed oral health status among older Hawaiian Chinese Americans [18]. Similarly, among native Spanish individuals, the immigrants were found to have worse oral health [19]. The oral health status of older Swedish immigrants was also lower than that of the native population of the same age [20]. Poor oral health was also observed among older Greek and Italian immigrants in Melbourne [21]. However, in China, studies regarding oral health of older immigrants have not been conducted so far.

Previous studies have shown that oral diseases are strongly associated with systemic diseases such as hypertension, diabetes, and cardiovascular disease [22,23]. A strong link between health and chronic diseases has also been found [24]. A study on elderly Mexicans showed that older adults with multiple chronic conditions had a higher prevalence of oral disease [25]. Hypertension was found to be associated with oral cancer in a nationwide population-based study conducted in Korea [26]. A survey in Hong Kong showed that nine chronic health conditions, including cancer, diabetes, hypertension, and stomach/intestinal diseases, were associated with oral health [27]. Furthermore, in a survey with older women, it was found that a history of inflammatory bowel disease was associated with poor oral health [28].

Migration is an extremely stressful phenomenon that produces significant levels of anxiety and depressive symptoms [29,30]. Extant literature in China has identified a significant increase in the prevalence of anxiety and depression among elderly migrants, which has become an important issue affecting their quality of life [31,32,33]. Previous studies have found that people with depression or anxiety are more likely to be at greater risk of dental disease, and that there is a positive correlation between depression and oral disease in older adults [34,35]. In a study on the relationship between psychological factors, oral health, and behavior, it was found that individuals with higher depression and anxiety scores had poorer periodontal health [36]. A Brazilian study showed that patients with depression had significantly lower GOHAI scores [37]. Furthermore, a Japanese study on older adults also showed the potential comorbidity of OHRQoL depressive symptoms and that depression impairs OHRQoL in older adults [38,39]. A study in Toronto found that individuals who perceived higher levels of stress also reported poor oral health [40].

To conclude, few studies have investigated the relationship between anxiety, depression, stress, and OHRQoL among middle-aged and older immigrants; no study has examined the relationships above in regard to MEFC. Therefore, this study aimed to explore the association between anxiety, depression, stress, morbidities, and OHRQoL among the MEFC in Weifang, China.

## 2. Materials and Methods

### 2.1. Data Collection and Research Participants

Data for this study were collected in Weifang, Shandong Province, China in August 2021. Individuals aged over 60 and following their children to Weifang had been recruited as participants. Multistage cluster random sampling was used to select the participants. In the first stage of data collection, four districts were chosen from the 12 districts as the primary sampling units (PSUs) while considering their economic development and geographic location. In the second stage, totally four sub-districts were selected from each of the PSUs as the secondary sampling units (SSUs). Finally, in the third stage, totally four communities were selected from each of the SSUs. All migrants who were older than 60 years and had followed their children to Weifang in these three communities made up the total sample of this study.

The formula used to calculate the sample size is as follows:

$$n=deff\frac{\mu^{2}\;p\left(1-p\right)}{\varepsilon^{2}}$$

In the formula above, the design efficiency was deff = 2.5, the level of confidence was μ = 1.96, the margin of error was ε = 10%, the prevalence of caries in the age group above 65 years old in the third national epidemiological survey of oral health status was *p* = 86.0%, and the non-response rate was 10% [41]. Based on the calculation of the above formula, it was determined that a minimum of 464 participants would be needed for the study.

After completing training on research background, questionnaire content, and social survey techniques, a total of 25 university students were assigned as research investigators for this study. The investigators conducted face-to-face interviews with each participant for approximately 20 min. Initially, 616 MEFCs were selected and interviewed. However, three participants were excluded as they answered their questionnaires incorrectly or incompletely. Ultimately, a total of 613 participants took part in the study, which was more than the required sample size of 464.

### 2.2. Measurements

#### 2.2.1. Oral Health-Related Quality of Life

The General Oral Health Assessment Index (GOHAI) scale was used to evaluate oral health-related quality of life. The GOHAI is a 12-question survey tool used to assess self-perceived oral health and explores the psychological impact of dental disease on pain, discomfort, dysfunction, and society. The GOHAI was determined using a 5-point Likert scale, with each point indicating the following: 1 = always, 2 = often, 3 = sometimes, 4 = rarely, and 5 = never. As the scale score is the sum of these values, a lower value indicates the presence of oral health problems. The overall GOHAI score, ranging from 12 to 60, was calculated for each individual, with higher scores indicating fewer oral problems and good OHRQoL [42]. In the analysis of this study, we used dichotomous categories of the GOHAI. Individuals with GOHAI scores of 12–56 were classified as the low-score group for oral health, while individuals with scores above 56 were classified as the high-score group [43,44]. The scale has also been validated in another elderly Chinese population [45].

#### 2.2.2. Social-Demographic Characteristics

The sociodemographic characteristics include gender, age, monthly income, educational level, years since migration, migration types, and reasons for migration. The ages of the participants were described using means and standard deviations. Other demographic characteristics were divided into sub-categories as follows: monthly income ((CNY 0–100 (USD 0–15.5), CNY 101–1000 (USD 15.6–154.5), CNY 1001–2000 (USD 154.7–309.2), CNY ≥ 2001 (USD ≥ 309.2)); educational level (Primary school education and below, Junior school education, Senior school education or above); years since migration (five years or less, and five years or more); types of migration (rural-to-urban migration and urban-to-urban migration); and reasons for migration (to take care of grandchildren, to get treatment for an illness, or rehabilitation, among others).

#### 2.2.3. Morbidities

Common diseases in the elderly that were included as part of the study comprised: hypertension, diabetes, cerebrovascular disease, gastrointestinal diseases, respiratory disease, and attendance at outpatient services. Participants had to indicate whether they had any of these diseases (yes or no) or if they had attended an outpatient dental service.

#### 2.2.4. Depression, Anxiety, and Stress

The researchers assessed the participants’ mental health using the Depression Anxiety Stress Scale (DASS-21), and the scores were calculated based on previous studies. The depression subscale consisted of questions 3, 5, 10, 13, 16, 17, and 21. The total depression subscale scores were divided into normal (0–9) and depression (10–42). The anxiety subscale consisted of questions 2, 4, 7, 9, 15, 19, and 20. The total anxiety subscale scores were divided into normal (0–7) and anxiety (8–42). The stress subscale consisted of questions 1, 6, 8, 11, 12, 14, and 18. The total stress subscale scores were divided into normal (0–14) and stress (15–42) [46]. DASS-21 has proven to be a reliable and effective measure for assessing the mental health of the Chinese population [47,48].

### 2.3. Statistical Analysis

The percentage of nominal variables were determined. Firstly, the chi-square test or *t*-test was used to explore the relationship between the variables of interest and oral health-related quality of life. Secondly, after a univariate analysis, all of the statistically significant variables were included in a logistic regression analysis. More specifically, the OHRQoL (assessed by the GOHAI score) was set as the dependent variable, while the independent variables included demographic characteristic-related variables, morbidity-related variables as well as depression, anxiety, and stress-related variables. Furthermore, the crude odds ratios (ORs) and 95% confidence interval (95% CI) were calculated. In Model 1, the independent variables included the basic demographic characteristic variables, Model 2 added morbidity-related variables to Model 1, while Model 3 added depression, anxiety, and stress-related variables to Model 2. All statistical analyses were performed using SPSS 26.0 (International Business Machines Corporation, Armonk, NY, USA), and statistical significance was set at *p* < 0.05.

## 3. Results

### 3.1. Demographic Characteristics

Table 1 presents the sociodemographic information of 613 MEFCs. Approximately 43.95% of the MEFCs were in the low-score group of the GOHAI. Of the participants, 73.1% were female and 26.9% were male, with an average age of 66.29 ± 5.518 years. Most of the MEFCs (83.4%) had a monthly income of less than CNY 2000 (≈USD 309.2). More than half of the MEFC had a primary school education or below (56.4%), 25.8% had a high school education, and only 17.8% had a high school education or above. A total of 56.4% of MEFC migrated less than five years ago, while the rest (43.6%) migrated five years ago. In addition, 85.6% of MEFC participants moved from rural to urban areas, while only 14.4% of the MEFCs moved from urban to urban areas. Furthermore, most MEFC migrated to take care of their grandchildren (86.9%), a small number migrated to get treatment for an illness or rehabilitation (2.9%), and the rest migrated for other reasons (10.1%). According to the chi-square analysis or *t*-test results, factors such as “age”, “educational level”, “years since migration”, and “migration type” were significantly correlated with GOHAI in MEFC.

### 3.2. Participants’ Morbidities

Table 2 shows the morbidities of the 613 MEFC participants. Common chronic diseases such as gastrointestinal disease (4.1%) were less common in MEFC. Furthermore, 28.7% of the MEFCs had hypertension and 8.8% of the MEFCs had diabetes, while only 27.4% of the MEFCs had attended an outpatient service in the past year. The chi-square test showed that “hypertension”, “gastrointestinal disease”, and “outpatient service attendance” were significantly correlated with GOHAI scores.

### 3.3. Depression, Anxiety, Stress, and GOHAI

Table 3 records the MEFC participants’ depression, anxiety, stress, and their GOHAI. Of the participants, 6.9% were depressed, 7.7% were anxious, and 3.5% were stressed. The chi-square test showed that depression, anxiety, and stress were significantly associated with the GOHAI score.

### 3.4. The Association between Morbidities, DASS, and GOHAI

To better demonstrate the relationship between morbidity, depression, anxiety, stress, and oral quality of life, we placed the results into three models using logistic regression (Table 4). In Model 1, we included only basic demographic information variables. The results showed that age, education, years since migration, and migration type were important predictors of the MEFC’s oral condition. When health condition variables were entered into Model 2, age, education, and migration type remained significant, but the significance of years since migration disappeared. Hypertension, gastroenteropathy, and outpatient services were also significant. When depression, anxiety, and stress were entered into Model 3, the significance of the migration type disappeared. Ultimately, the MEFC who were of older age (OR = 0.965, *p* = 0.039), with hypertension (OR = 0.567, *p* = 0.004), with gastroenteropathy (OR = 0.263, *p* = 0.007), received an outpatient service in the past year (OR = 0.669, *p* = 0.048), were depressed (OR = 0.338, *p* = 0.012), and anxious (OR = 0.414, *p* = 0.026) were less likely to report good oral health status. However, the MEFC with a high school education or above (OR = 1.872, *p* = 0.020) were more likely to report good oral health than those with primary school education and below.

## 4. Discussion

### 4.1. Association between Demographic Characteristics and OHRQoL

In this study, more than half of the participants (56.1%) were in the high-score group, which was defined as having good OHRQoL. The results of the fourth national oral health epidemiological survey showed that the level of oral health literacy of residents and the oral health of the elderly were gradually improving [49]. Moreover, the main reason for MEFC migration was to take care of grandchildren (86.9%); therefore, their health status was mostly good. This study found a statistically significant relationship between age, educational level, and oral health-related quality of life. Tooth loss increases with age [50], and oral diseases are becoming more common in the elderly population [51]. Therefore, it would seem that the older you are, the worse your oral condition could become. In addition, our findings suggest that MEFC with a high school education or above have better dental health-related quality than MEFC with an elementary school education and below, which is consistent with the results of previous studies [23,52]. The reasons may be that MEFC with higher educational levels may have more knowledge and awareness of oral protection strategies, pay more attention to oral hygiene, and seek medical attention more often if they contract oral diseases. Moreover, a previous study also found that parental socioeconomic status was associated with their children’s OHRQoL; that is, as parental socioeconomic status increased, their children’s OHRQoL increased as well [53].

This study found a correlation between the years since migration, migration type, and the GOHAI. Model 1 showed that MEFC who had migrated more than five years ago were less likely to report good oral health, possibly due to the fact that the elderly were not accustomed to the diet and water quality of the place they migrated to, which might have caused harm to their oral cavity. In addition, the MEFC people who engaged in urban-to-urban migration were more likely to report good oral health compared to those who engaged in rural-to-urban migration, which may be because of the good living conditions of the older people originally living in cities; since they pay more attention to oral cleaning and have more oral health knowledge, they have better tooth protection. However, after being entered into Model 3, the relation of migration age and migration type with the GOHAI was no longer significant. One possible reason for this is that morbidity, depression, anxiety, and stress have a greater impact on OHRQoL. This also provides a basis for evaluating OHRQoL in MEFC.

### 4.2. Association between Morbidities and OHRQoL

Consistent with previous studies, this research found that older adults with hypertension and gastrointestinal disease were less likely to report good oral health [54,55]. This may be because sick elderly people often take medication that harms the oral cavity or the stomach and is not good for the elderly diet. Furthermore, it also affects the oral cavity. In addition, many previous studies have shown that diabetes and cerebrovascular disease are also related to oral health [56,57,58,59], but this relationship was not significant in our study, which may be due to the low prevalence of diabetes (8.8%) and cerebrovascular disease (3.3%) among the MEFC sample in our survey. In addition, oral health is influenced by the health of the whole body [60]. Our study also found that older adults who had visited the clinic in the past year had worse oral health than those who had not, suggesting that other physical conditions can also affect oral health. This study also provides scientific evidence for promoting MEFC in the OHRQoL.

### 4.3. Association between Depression, Anxiety, Stress, and OHRQoL

This study found that depression and anxiety were strongly associated with OHRQoL. Depressed and anxious MEFCs were less likely to report good oral health, which is consistent with previous studies [61,62,63]. In addition, previous studies have shown a positive correlation between stress and poor oral health [40,64]. In our study, a significant relationship between stress and oral status was observed in the univariate analysis, and the significance disappeared after their inclusion in the model. This indicates that there is also an association between stress and oral health, but anxiety and depression have a greater effect on oral health in the MEFC than stress. Furthermore, this reflects a significant association between mental and oral health among the elderly. For elderly immigrants, their adaptability is often worse; especially for MEFC, the focus of their lives is often on taking care of their grandchildren, which may lead them to neglect their physical and mental health problems. Moreover, due to the new living environment and new neighbors, they are more likely to have psychological problems, which may also have a negative impact on oral health.

### 4.4. Implications

In view of the above findings, morbidities, depression, and anxiety were key factors affecting the oral health of MEFCs. To improve the OHRQoL in MEFCs, targeted effective measures should be taken. Firstly, the children of the MEFC should spend more time with the MEFC to lower their psychological tension in the new environment as well as encourage and help the MEFC to shift to a healthier lifestyle with regard to oral health after the MEFC’s arrival at new cities from the rural areas. Secondly, the MEFC should be open and ready to adapt to the life of the inflow city. Thirdly, for the community and government, free medical checkup programs and health promotion activities should be offered to the MEFCs to increase their knowledge on chronic diseases and oral health. Fourthly, more recreational activities could be conducted to increase the social network and lower the depression, anxiety, and stress of MEFC after their coming to the new cities.

### 4.5. Limitations

This study had some limitations. Firstly, this study used a cross-sectional design; therefore, it could not predict the causal relationship between OHRQoL and morbidity, depression, anxiety, and stress. Secondly, due to the COVID-19 pandemic, our survey in Shanghai was not conducted as scheduled; therefore, the data were limited to the Weifang area, which may not be representative of the MEFC in China. Thirdly, data collection was completed by self-reporting, which may have made the recall and reporting bias inevitable.

## 5. Conclusions

It was found that 43.9% of MEFCs had poor OHRQoL, and 56.6% of MEFCs had good OHRQoL. The MEFC who were of older age, with hypertension, with gastroenteropathy, had received outpatient service in the past year, were depressed as well as anxious were less likely to report good OHRQoL. In contrast, the MEFC with higher education were more likely to report good OHRQoL. As the first study to investigate the determinants of OHRQoL among Chinese MEFC from the perspective of morbidities, depression, anxiety, and stress, the results indicate that the fewer morbidities, the lower the level of depression and anxiety and the better the OHRQoL of the MEFC.

## Figures and Tables

**Table 1:** Descriptive analysis of the demographic characteristics of the MEFC by GOHAI.

Variables	Total	Low GOHAI Group (GOHAI ≤ 56)	High GOHAI Group (GOHAI ≥ 57)	χ^2^/t	*p*
Observations	613 (100)	269 (43.9)	344 (56.1)		
Age	66.29 ± 5.518	67.07 ± 6.042	65.67 ± 4.992	3.153 ^a^	0.001
Gender					
Male	165 (26.9)	68 (25.3)	97 (28.2)	0.654 ^b^	0.419
Female	448 (73.1)	201 (74.7)	247 (71.8)		
Monthly income					
CNY 0–100 (USD 0–15.5)	210 (34.3)	100 (37.2)	110 (32.0)	2.603 ^b^	0.457
CNY 101–1000 (USD 15.6–154.5)	234 (38.2)	99 (36.8)	135 (39.2)		
CNY 1001–2000 (USD 154.7–309.2)	67 (10.9)	25 (9.3)	42 (12.2)		
CNY ≥ 2001 (≥USD 309.2)	102 (16.6)	45 (16.7)	57 (16.6)		
Educational level					
Primary and below	346 (56.4)	172 (63.9)	174 (50.6)	13.700 ^b^	0.001
Junior school	158 (25.8)	64 (23.8)	94 (27.3)		
Senior school or above	109 (17.8)	33 (12.3)	76 (22.1)		
Migration years					
5 years and below	346 (56.4)	137 (51.0)	209 (60.8)	5.929 ^b^	0.015
Above 5 years	267 (43.6)	132 (49.0)	135 (39.2)		
Migration type					
Rural to urban	525 (85.6)	241 (89.6)	284 (82.6)	6.073 ^b^	0.014
Urban to urban	88 (14.4)	28 (10.4)	60 (17.4)		
Migration reason					
Taking care of grandchildren	533 (86.9)	228 (84.8)	305 (88.7)	4.270 ^b^	0.118
Curing a disease or rehabilitation	18 (2.9)	12 (4.4)	6 (1.7)		
Others	62 (10.1)	29 (10.8)	33 (9.6)		

Note: GOHAI = Geriatric Oral Health Assessment Index; ^a^ = *t*-test, ^b^ = Chi-square test.

**Table 2:** Morbidities and GOHAI of the MEFC.

Variables	Total	Low GOHAI Group (GOHAI ≤ 56)	High GOHAI Group (GOHAI ≥ 57)	χ^2^	*p*
Observations	613 (100)	269 (43.9)	344 (56.1)		
Hypertension					
No	437 (71.3)	170 (63.2)	267 (77.6)	15.334	<0.001
Yes	176 (28.7)	99 (36.8)	77 (22.4)		
Diabetes					
No	559 (91.2)	242 (90.0)	317 (92.2)	0.900	0.343
Yes	54 (8.8)	27 (10.0)	27 (7.8)		
Gastrointestinal disease					
No	588 (95.9)	250 (92.9)	338 (98.3)	10.917	0.001
Yes	25 (4.1)	19 (7.1)	6 (1.7)		
Outpatient service attendance					
No	445 (72.6)	175 (65.1)	270 (78.5)	13.691	<0.001
Yes	168 (27.4)	94 (34.9)	74 (21.5)		

**Table 3:** Depression, anxiety, stress, and the GOHAI of the MEFC.

Variables	Total	Low GOHAI Group (GOHAI ≤ 56)	High GOHAI Group (GOHAI ≥ 57)	χ^2^	*p*
Observations	613 (100)	269 (43.9)	344 (56.1)		
Depression					
No	571 (93.1)	237 (88.1)	334 (97.1)	19.112	<0.001
Yes	42 (6.9)	32 (11.9)	10 (2.9)		
Anxiety					
No	566 (92.3)	234 (87.0)	332 (96.5)	19.337	<0.001
Yes	47 (7.7)	35 (13.0)	12 (3.5)		
Stress					
No	592 (96.6)	255 (94.8)	337 (98.0)	4.584	0.032
Yes	21 (3.4)	14 (5.2)	7 (2.0)		

**Table 4:** Binary logistic regression of demographic characteristics, morbidities, depression, anxiety, stress, and GOHAI of the MEFC.

Variables	Model 1			Model 2			Model 3		
	OR	95% CI	*p*	OR	95% CI	*p*	OR	95% CI	*p*
Age									
	0.960	0.930, 0.991	0.012	0.858	0.927, 0.991	0.012	0.965	0.933, 0.998	0.039
Educational level									
Primary school and below									
Junior school	1.223	0.824, 1.815	0.318	1.083	0.721, 1.626	0.702	1.114	0.736, 1.686	0.610
Senior school or above	1.733	1.054, 2.850	0.030	1.727	1.036, 2.879	0.036	1.872	1.105, 3.173	0.020
Migration years									
5 years and below									
Above 5 years	0.712	0.510, 0.996	0.047	0.794	0.562, 1.122	0.191	0.790	0.555, 1.124	0.190
Migration type									
Rural to urban									
Urban to urban	1.791	1.051, 2.850	0.032	1.862	1.077, 3.219	0.026	1.745	0.998, 3.052	0.051
Hypertension									
No									
Yes				0.555	0.380, 0.811	0.002	0.567	0.386, 0.935	0.004
Gastrointestinal disease									
No									
Yes				0.261	0.099, 0.689	0.010	0.263	0.099, 0.699	0.007
Outpatient service attendance									
No									
Yes				0.604	0.411, 0.888	0.010	0.669	0.450, 0.996	0.048
Depression									
No									
Yes							0.338	0.145, 0.789	0.012
Anxiety									
No									
Yes							0.414	0.191, 0.901	0.026
Stress									
No									
Yes							0.909	0.310, 3.157	0.985

Note: Dependent variable = GOHAI score, with 0 = low GOHAI scores and 1 = high GOHAI scores; Model 1, the independent variables include the basic demographic characteristic variables, Model 2 added morbidity-related variables to Model 1, Model 3 added depression, anxiety, stress-related variables to Model 2.

## Data Availability

The datasets used and analysed in this study are available from the corresponding author on reasonable request.
